# Caries prevalence among 18 years old, an epidemiological survey in Israel

**DOI:** 10.1186/s13584-020-00402-4

**Published:** 2020-08-31

**Authors:** Nirit Yavnai, Sigal Mazor, Yuval Vered, Idan Shavit, Avraham Zini

**Affiliations:** 1 IDF Medical Corps Headquarters, Military Post #02149, IDF, Tel Hashomer, Ramat Gan, Israel; 2 Department of Community Dentistry, Faculty of Dental Medicine, Hebrew University, Hadassah Ein Kerem Campus, Jerusalem, Israel; 3 Vice Dean and the Department of Community Dentistry, Hebrew University-Hadassah School of Dental Medicine, Hadassah Ein Kerem Campus, Jerusalem, Israel

**Keywords:** Caries, Prevalence, 18 years old

## Abstract

**Background:**

There is a lack of evidence on caries prevalence among 18 years old Israeli young adults with only a scarce evidence regarding this index age group. In the last few years dental care policy in Israel underwent substantial changes and a major reform in dental services was led by the Israeli Ministry of Health, including coverage of dental care for children by the state. In addition, a cessation of community water fluoridation was in a debate.

The objective of the current study was to describe prevalence of caries among 18 years old Israeli young adults and to evaluate possible associations with personal and demographic variables.

**Methods:**

The study was a cross sectional clustered survey. Participants were recruited to the study at their first day of military service. Participants completed a questionnaire for personal and demographic data, including: age, country of birth, education, and current smoking status. Then participants underwent clinical evaluation included DMFT and caries free rates. No radiographic evaluation was included in the current study. Univariate and multivariate statistical analysis were performed.

**Results:**

A total of 702 participants were included in the study, 58.4% were males. Their mean age was 19.03 ± 0.65 years, 91.3% of the participants were born in Israel. Mean DMFT was 1.95 ± 2.67, and 46.7% (*n* = 328) were caries free. Higher DMFT score was significantly associated with participant’s parents’ education, country of birth, and smoking status. Lower caries free rates were significantly associated with participant’s parents’ education, and smoking status. After linear regression for total DMFT, all variables were significant predictors to higher DMFT, except father’s education, while logistic regression for caries free, only mother’s education was found to be a significant predictor.

**Conclusions:**

The current study presents encouraging low DMFT levels. Participants in this study were not included in the dental care services reform, and did enjoy the benefits of water fluoridation, enabling the results to play an important baseline data for future reference. Additionally, results should be considered when planning intervention programs for at risk groups.

**Trial registration:**

This study was registered in ClinicalTrials.gov (Identifier No. NCT02958891, November 8th, 2016) and was approved by the IDF Institutional Review Board (#1524–2015).

## Background

The last nation-wide significant-extent dental epidemiological survey was published almost two decades ago, describing caries prevalence in Israel among 21 years old military personnel at their day of discharge from the mandatory military service [1]. Some additional scarce evidence regarding caries prevalence among 18 years old in Israel were found in a research aimed to evaluate association between caries prevalence and black extrinsic discoloration [2]. Mean DMFT was 4.2–5.98 (with standard deviation of 3.9–4.8) for a wide range inhomogeneous age group of 18–29-year-old participants. In a very local survey, mean DMFT among 18–19 Israeli military recruits was 6.09 ± 5.29 with 17.2% caries free rate [3]. Since these publications, no representative national epidemiological survey regarding caries prevalence in Israel among 18 years old has been published yet, nor did any additional study of caries prevalence among this age group, although this age group is defined by the World Health Organization (WHO) as an index group.

Prior to 2010, besides specific primary prevention program given at primary schools, dental treatment for children and adolescents in Israel was the sole responsibility of their parents and was paid in a largely privately funded delivery system [4]. In the last few years dental care policy in Israel underwent substantial changes [5]. The major and significant reform occurred in 2010 with the inclusion of dental care in the National Health Insurance Law services’ basket, by covering dental care to children by the state [4–6]. This reform was performed gradually, starting by including dental treatment to children from birth to 8 years old, and was eventually broadened to the age 18 nowadays.

The rationale behind this reform was to cover all basic dental treatment needs for children until one enters the mandatory military service at the age of 18, where he is eligible to receive most of his dental treatment needs in the military setting.

In addition, Community water fluoridation was the main prevention policy of the Israeli Ministry of Health for many years. In recent years, there have been some changes in this policy in Israel [7], and in 2014 the policy was changed, and community water fluoridation was stopped [4]. In 2016 the Ministry of Health had reapproved regulations and renewal of water fluoridation policy, but, due to procedural and technical issues, water fluoridation is still not renewed.

The purpose of this study was to describe prevalence of caries among 18 years old military recruits and to evaluate possible associations with personal and demographic variables. The results of this survey would be helpful for service planning and for future reference, in order to evaluate the effect of the reform in dental services and the cessation of water fluoridation on dental health in Israel.

## Methods

The study was a cross sectional clustered survey. The sampling frame was all military recruits in 2016. Specific recruitment days were chosen for data collection as clusters, to enable representation to all military professions for both genders along 2016 at the solely unique recruitment base of the Israel Defense Forces (IDF) in the center of Israel. Participants were recruited to the study at their first day of military service. The data was collected at the military recruitment process that every military recruit must undergo, between March and August 2016. Inclusion criteria were military recruits, at their first day of military service, from both genders, 18 or more years old. Exclusion criteria were having chronic bone diseases, any skeleton development defects or calcification impairments in calcified tissues.

Sample size was calculated using Raosoft website [8] with the following assumptions: confidence level of 99% (α = 1%), response rate of 50%, assumed population size of 100,000 (based on the Israeli Bureau of Statistics data) and an in-study attrition rate of 5%. The total sample size was 700 participants.

After signing an informed consent form, participants completed a self reporting questionnaire collecting data regarding demographic and personal data including age, country of birth, participant and parents’ education (elementary/high school/has a matriculation diploma/academic) and current smoking status (yes/no). Then participants had a clinical examination by one of the five study dentists (N.Y., S.M., Y.V., I.S. and A.Z.). Calibration between the study dentists was performed prior to data collection days. Twenty different cases were evaluated and discussed by all five certified dentists who were the researchers in this study (Kappa = 0.7–0.8).

Caries history was evaluated by Decayed, Missing, Filled Tooth index (DMFT) for all available permanent teeth. Wisdom teeth were excluded. The evaluation was performed according to the WHO instructions for caries diagnosis for epidemiological surveys, using a dental mirror and a WHO dental probe for caries detection [9].

Mean DMFT score was calculated for each participant. All dental measurements were performed using standard office chair with office mixed with natural window illumination. No dental unit was involved, neither dental light nor triple syringe. No radiographic evaluation was included in the current study.

Statistical analysis included Chi square and t tests for univariate analyses. A linear regression and logistic regression models were built for multivariate analyses. Multivariate models included significant variables from the univariate analyses, additionally to age and gender adjustment. Level of significance was set to *P* < 0.05.

The study was approved by the IDF Institutional Review Board (IRB) (#1524–2015) and was conducted in full accordance with all ethical principles.

## Results

A total of 702 participants were included in the study. Of them, 58.4% were males, mean age was 19.03 ± 0.65 years. Almost all of the participants (91.3%) were born in Israel. Most of the participants (80.1%) had finished high school and were eligible for a matriculation diploma. Parents’ educational status showed that most of participants’ parents did not have an academic degree. A fifth (19.9%) reported current smoking. Additional personal data is presented in Table 1.

**Table 1:** Distribution of personal and demographic data with participants’ DMFT^a^ levels and caries free prevalence

	N (%)	DMFT (SD^b^)	*P* value (t test)	Caries Free N (%)		*P* value (χ^2^ test)
				***No***	***Yes***	
**Gender**			0.879			0.889
**Male**	395 (58.4)	1.95 (2.75)		213 (53.9)	182 (46.1)	
**Female**	281 (41.6)	1.97 (2.55)		150 (53.4)	131 (46.6)	
**Participant’s Education (has matriculation diploma)**			0.090			0.132
**Yes**	559 (80.1)	1.86 (2.63)		290 (51.9)	269 (48.1)	
**No**	139 (19.9)	2.29 (2.82)		82 (59.0)	57 (41.0)	
**Mother’s Education**			< 0.001			0.002
**Academic**	322 (47.7)	1.49 (2.23)		213 (53.9)	182 (46.1)	
**Else**	353 (52.3)	2.30 (2.86)		150 (53.4)	131 (46.6)	
**Father’s Education**			0.002			0.010
**Academic**	297 (45.2)	1.55 (2.38)		207 (57.5)	153 (42.5)	
**Else**	360 (54.8)	2.18 (2.72)		141 (47.5)	156 (52.5)	
**Country of Birth**			0.013			0.679
**Israel**	640 (91.3)	1.87 (2.61)		339 (53.0)	301 (47.0)	
**Other**	61 (8.7)	2.75 (3.16)		34 (55.7)	27 (44.3)	
**Current Smoker**			0.003			0.013
**Yes**	139 (19.9)	2.55 (3.11)		87 (62.6)	52 (37.4)	
**No**	560 (80.1)	1.80 (2.54)		285 (50.9)	275 (49.1)	
**Total**		**1.95 (2.67)**		**374 (53.3)**	**328 (46.7)**	

^a^Decayed, Missing or Filled Teeth

^b^Standard Deviation

Mean DMFT was 1.95 ± 2.67 (D = 0.52 ± 1.19; M = 0.03 ± 0.17; F = 1.40 ± 2.32). Higher DMFT score was significantly associated with poor education of participants’ parents (*P* < 0.001 for mother’s education; *P* = 0.002 for father’s education), born outside of Israel (*P* = 0.013) and a current smoker (*P* = 0.003) (Total DMFT results are presented in Table 1). Age was significantly correlated with DMFT (Pearson coefficient = 0.087; *P* = 0.021).

Caries free rate was 46.7% (*n* = 328) among the participants. Lower caries free rates were significantly associated with poor education of participants’ parents (*P* = 0.002 for mother’s education; *P* = 0.010 for father’s education), and being a current smoker (*P* = 0.013). Caries free rates were not significantly associated with age (*P* = 0.106) (Table 1).

After linear regression for total DMFT (R^2^ = 0.056; *P* < 0.001), country of birth, age, mother’s education, and smoking status were significant predictors to higher DMFT (Table 2). After multivariate logistic regression (Table 3) only mother’s education was a significant predictor to caries free (Nagelkerke R^2^ = 0.045; *P* = 0.002).

**Table 2:** Multiple Linear Regression Model for significant independent variables effect on total DMFT^a^ (*P* < 0.001, R^2^ = 0.065) (adjusted for gender and age)

	B	Beta	*P* value	Confidence Interval
Constant	−5.532	–	0.066	−11.438 - 0.373
Country of Birth (Israel vs.^b^ Other)	−1.323	−0.137	< 0.001	−2.065 - -0.581
Gender (Male vs. Female)	0.199	0.038	0.352	−0.220 – 0.618
Age (Continuous variable)	0.468	0.120	0.003	0.162–0.774
Mother’s Education (Academic vs. Else)	−0.735	−0.142	0.005	−1.250 – − 0.220
Father’s Education (Academic vs. Else)	−0.179	− 0.034	0.503	− 0.702 – 0.345
Current Smoker (Yes vs. No)	0.563	0.084	0.040	0.026–1.101

^a^Decayed, Missing or Filled Teeth

^b^versus

**Table 3:** Multivariate Logistic Regression Model for significant independent variables effect on caries free among 18 years old Israeli recruits (*P* = 0.027, Nagelkerke R^2^ = 0.045) (adjusted for gender and age)

	B	*P* value	OR^a^	Confidence Interval
Constant	4.962	0.050	142.879	–
Country of Birth				
Other	–	–	1.000	–
Israel	0.377	0.228	1.458	0.790–2.692
Mother’s Education				
Else	–	–	1.000	–
Academic	0.425	0.045	1.529	1.010–2.315
Father’s Education				
Else	–	–	1.000	–
Academic	0.174	0.417	1.190	0.782–1.813
Current Smoker				
No	–	–	1.000	–
Yes	−0.378	0.093	0.685	0.440–1.066

^a^Odds Ratio

## Discussion

This study analyzed data regarding caries experience and demographic variables of seven hundred and two 18 years old Israeli military recruits on their first day of military service. Its predominant advantage is the representativeness of the sample which is optimally representative of Israeli young healthy adults. The distribution of genders is similar and representative of the recruitment gender rate among Israeli youth.

This study presents some unexpected and encouraging findings of low DMFT scores and high caries free rates comparing to previous published data [1–3]. Data among this age group from the world presented higher DMFT scores, though the trend of decrease was similar [10–12].

The epidemiology of dental diseases has clearly been described as a web of connecting factors, including biological, social, psychological, economic, environmental and other variables [13] and many socio-demographic markers were already been shown to be associated with caries prevalence [14]. Previous studies conducted in Israel showed significant associations between education, country of birth, smoking and caries experience [1, 14, 15]. Additionally, Levy et al. had showed a statistically significant association between low intellectual capabilities and higher dental treatment needs among IDF soldiers, and implied for an association with parental education, similarly to other published studies and to the current study results [14, 16, 17]. Parental education level had been previously found to be associated with oral health outcomes, and specifically, with caries prevalence in many studies [18–20]. The odds of having any caries experience (DMFT > 0) were found to be significantly greater in those with low parental educational or occupational background [21], thus, the results of the current study support former evidence.

Additionally, current smoking was found as a significant predictor to higher DMFT. These results are similar to the scientific literature and the association between smoking and caries prevalence is well established in the literature [22, 23].

Caution should be taken with these findings since although military service is mandatory in Israel, there are two communities (Jewish Orthodox and Arabs) that are partially exempt from military service. However, these communities were not represented in former surveys, allowing comparability with previous studies conducted in the Israeli military setting.

It is important to mention that using the DMFT index for evaluation of caries prevalence has some well-known limitations: it is based on clinical evaluation without X-ray imaging, thus might underestimates caries prevalence [24]; has some potential inter-observer bias and variability [25]; gives equal weight to untreated decay, missing and filled teeth; does not refer to reasons for tooth loss [26]; and does not provide useful treatment needs estimation [27].In the last decade, the Israeli Ministry of Health has led and implemented a major reform and expanded the prevention and treatment programs in the dental health services, mainly for children and adolescents [4, 5]. In the near future, with these programs implemented, soldiers should need less dental treatment [4]. The current study was performed among 18 years old youngsters who were not included in the dental care services reform, and did enjoy the benefits of water fluoridation during their adolescence years, enabling the results to play an important baseline data for future reference to assess the effect of these significant changes. We suggest that future evaluation studies for the efficacy of the Israeli dental services reform might possibly find different F to D components ratio within the DMFT scores.

The current study with the presented low DMFT levels should help monitoring dental health among this age group while the Israeli dental care services reform is proceeding. According to our results, some suggested risk factors including, being a current smoker, not being born in Israel and with low parental education may be associated with higher DMFT levels. Nevertheless, the encouraging results of caries prevalence may suggest to the Ministry of Health dental services, and specifically to the IDF dental department, to consider focused comprehensive preventive programs, while using these supporting scientific evidence to refine at risk populations (males, current smokers, from immigrant families with low parental education), while continue monitoring caries prevalence among all groups.

## Conclusions

The results enabling national and international comparisons of caries prevalence within this important index group. In addition, the results can offer a unique opportunity to explore the Israeli dental health services reform and perform an evidence-based evaluations and substantial national follow up survey after the community water fluoridation cessation. In Addition, results should be considered when planning prevention intervention programs for designated at risk groups, such as current smokers or for those who were not born in Israel, since the IDF has some unique capabilities to outreach specific selected populations.

## Data Availability

The datasets used and analyzed during the current study are available from the corresponding author on reasonable request.

## Abbreviations

IDF: Israel Defense Forces
WHO: World Health Organization
DMFT: Decayed, Missing, Filled Tooth
IRB: Institutional Review Board
